# Future Shorelines: A Living Shoreline Site Selection and Design Decision Support Tool that Incorporates Future Conditions Induced by Sea Level Rise

**DOI:** 10.1007/s12237-024-01425-9

**Published:** 2024-09-04

**Authors:** Randall W. Parkinson, Levente Juhasz, Jinwen Xu, Zhaohui Jennifer Fu

**Affiliations:** 1 Institute of Environment, Florida International University, Miami, FL 33199 USA; 2 Geographic Information Systems Center, Florida International University, Miami, FL 33199 USA; 3 Present Address: Institute of Transportation Studies, University of California, Berkeley, CA 94720 USA

**Keywords:** Climate change, Coastal resiliency, Environmental justice, Florida, Living shorelines, Indian River Lagoon, Nature-based solutions, Sea level rise

## Abstract

Most living shoreline site selection and design decision support tools are based upon existing environmental conditions. We developed a web-based, geospatial tool called Future Shorelines that integrates high-resolution landscape elevation data and a matrix of locally derived NOAA Interagency Sea Level Rise Scenarios to characterize future conditions of submergence and shoreline translation induced by sea level rise. Once the practitioner selects a location of interest, sea level rise scenario (e.g., high), and target year (e.g., 2050), the tool will generate plan view and cross-sectional informational graphics specific to their choices. This information can then be paired with other menu options, like parcel ownership, to facilitate the planning and construction of nature-based shoreline stabilization solutions that (1) are located where opportunities for horizontal migration are optimized, (2) remain accessible for monitoring and maintenance, and (3) perform as intended over the design life of the installation. The tool’s menu options and the user interface were informed by project partner input solicited during numerous workshops convened over the duration of the 2-year project. This coproduction created a product that was familiar to the end user and therefore increased the likelihood that it would be utilized by them during the planning and design of living shoreline projects. Although developed for use in the Indian River Lagoon, located along the east-central Florida coast, it can be seamlessly replicated for application in other coastal regions of the USA where the requisite data are available.

## Introduction

Over the last century, there has been a proliferation of shoreline stabilization projects constructed along the developed coastlines of the USA to mitigate the effects of coastal erosion and flooding (Miller et al. 2016). Initially, these consisted of bulkheads, seawalls, and revetments collectively described as hard structures (Swann 2008; NOAA 2015). Within the past several decades however, it became evermore apparent to coastal practitioners that the cumulative effect of their construction was a substantial loss of natural habitat and the ecosystem services they provided (Broome, et al. 1992; Currin et al. 2010; Miller et al. 2016). As a result, a new strategy emerged that advocated for the use of natural materials or nature-based solutions (e.g., living shorelines) as a means of mitigating erosion (National Research Council 2007; Mitsova and Bergh 2016; Bilkovic and Mitchell 2017; Reguero et al. 2018; Gijsman et al. 2021; van Hespen et al. 2022; Temmerman et al. 2023). As the use of living shorelines (aka soft or green structures) expanded, so too did the need for the development of technical guidelines to facilitate effective (1) site selection, (2) installation design, and (3) performance monitoring (Restore America’s Estuaries 2015).

To date, most living shoreline site selection and design decision support tools are based upon existing environmental conditions (e.g., shoreline type, presence of structures, habitat, wind/wave exposure) (Berman and Rudnicky 2008; Mitsova and Bergh 2016; Zylberman 2016; Kibler et al. 2020; Nunez et al. 2022; Boyd et al. 2024). However, these conditions will change in response to the effects of a warming climate and concomitant sea level rise. Along the Florida coast, for example, sea level is expected to rise ~ 0.3 m above present (2020) by 2050 (NOAA 2022). This will result in coastal flooding and shoreline translation. Yet very few living shoreline site suitability models consider future conditions in their analysis (cf. Balasubramanyam and Howard 2019) and none utilize locally derived sea level scenarios or parcel-specific data.

The goal of this project was to create a user-friendly, web-based geospatial living shoreline decision support tool for coastal practitioners that models future conditions (e.g., submergence, shoreline change) induced by sea level rise (Table 1). This information can then be paired with other menu options (e.g., parcel ownership and value) to facilitate the planning and construction of nature-based shoreline stabilization solutions that (1) are located where opportunities for horizontal migration are optimized, (2) remain accessible for monitoring and maintenance, and (3) perform as intended over the design life of the installation. To demonstrate the tool’s functionality and utility, four examples of its output are described.

**Table 1:** Summary of living shoreline site suitability decision support attributes considered in existing models and those included in this study (novel). Also shown are next generation attributes requested by project partners to facilitate compliance with state statutes and permitting

Approach	Status	Attribute	Rationale
Existing	NA	Shoreline type, presence of structures	Assessment of existing environmental conditions
		Shoreline/nearshore slope	
		Habitat/substrate type	
		Wave climate	
Novel	This study	Shoreline transgression	Assessment of future conditions
		Upland topographic slope	Assessment of capacity to migrate landward
		Future land use/ownership	Assessment of recruitment capacity
		Demographics	Assessment of group representation
		Shoreline/upland characteristics	Assessment of capacity to migrate landward
	Next generation	State of Florida sea level rise scenarios	Requested by project partners
		Alternate demographic indices	
		Bathymetry	
		Muck deposits	
		Sea grass distribution	
		Stormwater outfalls	
		Impoundment culverts	
		Alternate tidal datums	
		Other shoreline or environmental data	

## Area of Study and Background

**Fig. 1:**
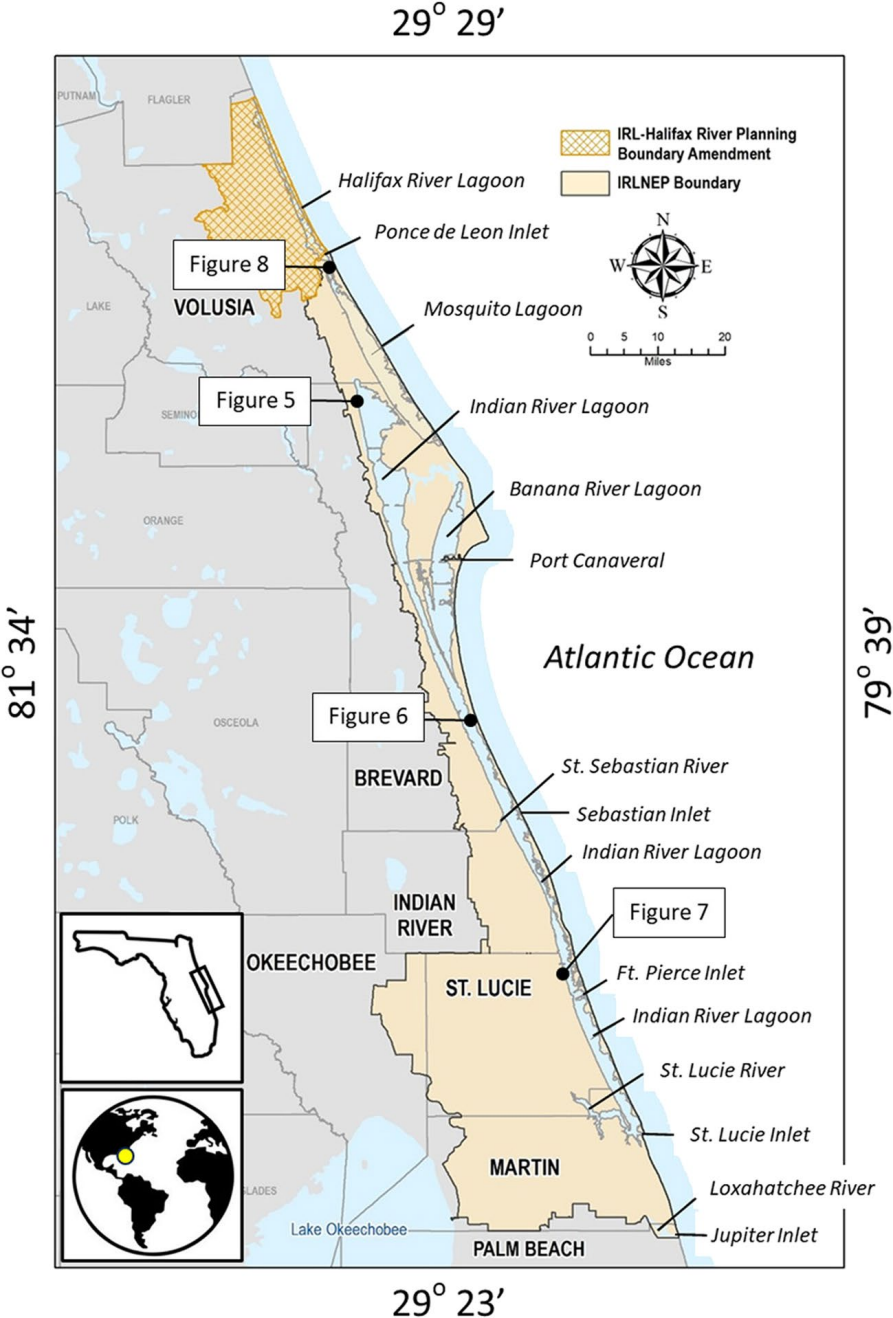
Regional location map and approximate geographic boundaries of the study domain (Indian River Lagoon). Also shown are the counties located within the watershed and other figure locations referenced in text

This project focused on the Indian River Lagoon (IRL) (Fig. 1), a 250-km-long shore-parallel micro-tidal estuary located within the east-central Florida barrier island complex. It covers an area of 353 km^2^ and is composed of three distinct and connected water bodies: the Indian River, Banana River, and Mosquito Lagoon. Water depths average about 2 m and historically the bottom was covered by extensive seagrass that has since declined within all three basins as a consequence of the deterioration of water clarity (Steward et al. 2005). Most of the IRL’s tidal wetlands were filled, ditched, or impounded over the past century (Brockmeyer et al. 2021). Those that remain are located primarily in land conservation areas (e.g., Merritt Island National Wildlife Refuge) or mosquito control impoundments. In the southern and central portion of the IRL, the wetlands consist of mangrove-dominated plant communities that transition northward into salt marsh. Its 2284 km^2^ humid subtropical watershed sprawls over five coastal counties. The lagoon’s total annual economic contribution to the region is estimated to be about ten billion dollars and generated in part by the ecosystem services it provides, including recreational fishing and ecotourism (East Central Florida Regional Planning Council and Treasure Coast Regional Planning Council 2016). Hence, there is keen interest in restoring and sustaining the ecological value and function of the IRL.

**Fig. 2:**
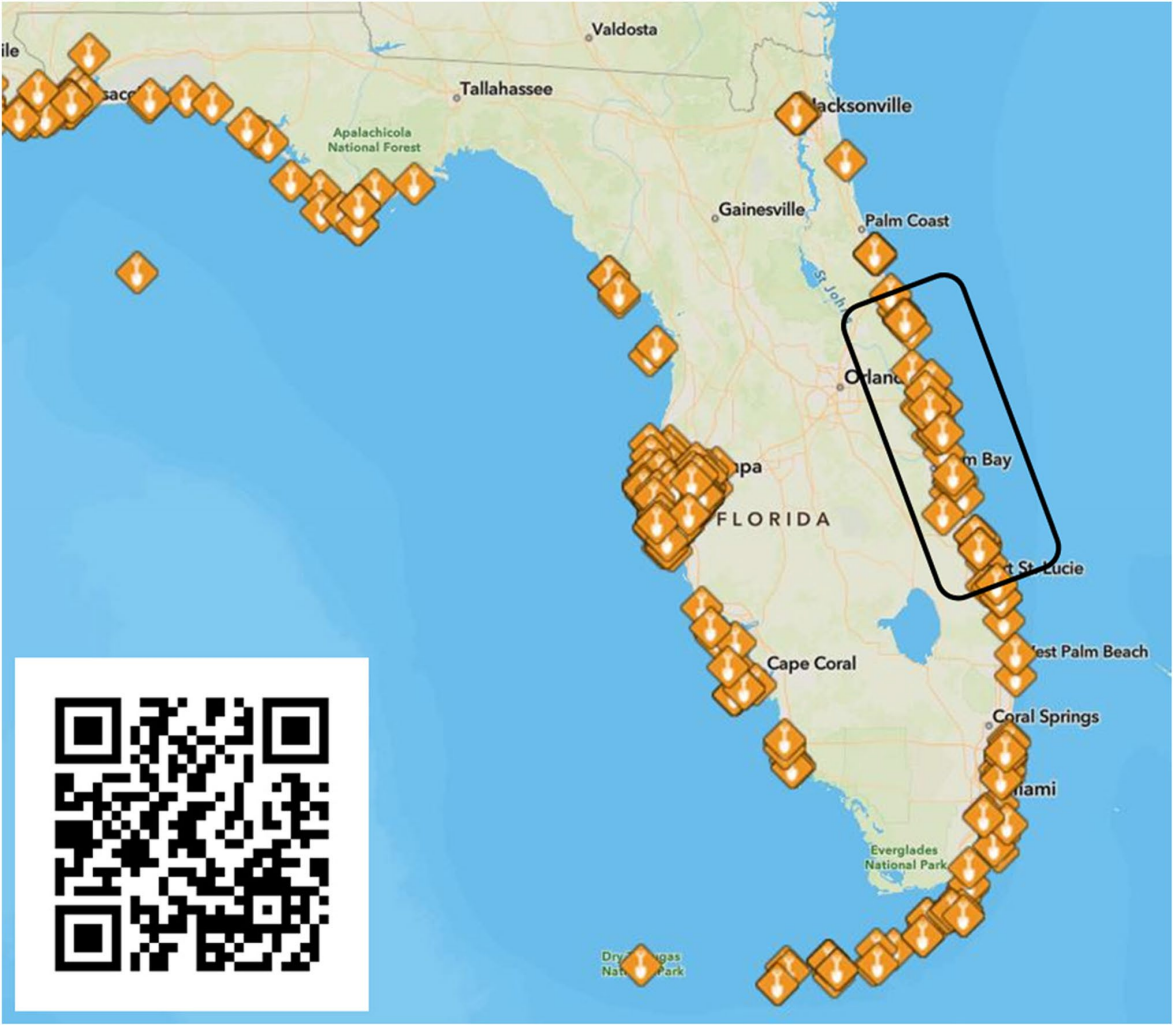
Screen grab of the NOAA Restoration Atlas (NOAA 2024) indicating location of more than 200 nature-based coastal restoration projects within the State of Florida. Black boxed inset is the study domain of this project. Inset is Future Shorelines QR code

The IRL was recognized by the Environmental Protection Agency (EPA) as an Estuary of National Significance in 1990 and is one of 28 National Estuary Programs (NEP). Since then, a growing body of scientific evidence revealed that the ecological and biological integrity of the lagoon had degraded over historical times due to a decline in water quality (Sigua et al. 2000; Lapointe et al. 2015) caused by watershed urbanization (e.g., stormwater runoff, septic systems). The IRL NEP and all coastal counties within the watershed now have extensive programs designed to mitigate water quality impairment, including (1) septic-to-sewer conversions, (2) stormwater retrofits (e.g., baffle boxes and retention-detention basins), and (3) living shorelines (cf. Tetra Tech, Inc. 2022). Because living shorelines benefit from broad public support and are perceived as a cost-effect means of improving the health of the lagoon (Parkinson 2023), a large and rapidly growing number of projects have been completed or are planned within the watershed and throughout the Florida coastal zone (Fig. 2) using funds provided by numerous federal (e.g., NOAA, EPA, NPS), state (e.g., Florida Fish and Wildlife Commission), and local (e.g., Brevard County Save our Indian River Lagoon) resource regulatory and management agencies.

Two recent studies (Parkinson and Seidel 2018; Parkinson et al. 2020) conducted on behalf of the IRL NEP determined increasing impairment of water quality is highly likely under conditions of future climate change and concomitant sea level rise. Specific to living shorelines, the logical questions to ask given this prognosis are as follows: (1) how will the ecosystem services (e.g., nutrient uptake, shoreline stabilization, and a reduction in sediment pollution) of living shorelines change in response to sea level rise and (2) can a priori knowledge of shoreline changes induced by rising seas be utilized during the process of site selection and design to enhance their resilience? This project was designed to bolster the capacity to answer question 2.

## Methods

### Data Collection and Processing

The full suite of data used to model future conditions along the IRL shoreline is summarized in Table 2. Four NOAA (Sweet et al. 2022) scenario-based sea level rise trajectories (i.e., intermediate-low, intermediate, intermediate-high, and high) and time steps (2030, 2040, 2050, and 2100) were considered in this project. The NOAA trajectories are bounded by a simple linear extrapolation of sea level rise since the early 1990s (low; not considered in this study) to a rise representing extreme ice-sheet melting and discharge (high). We extracted the median values corresponding to each scenario as calculated at the Trident Pier (Port Canaveral, FL) station using the NASA Interagency Sea Level Rise Scenario Tool (NOAA 2022).

**Table 2:** Summary of the source and application of the datasets utilized in the Future Shorelines tool to model future conditions along the IRL shoreline

Dataset	Source	Application
Sea level rise scenarios	NOAA (2022)	Inundation mapping Future shoreline location
Digital elevation model	Florida Peninsular LiDAR Survey 2018–2020 Florida Div. of Emerg. Mgmt. (OMC Partners 2024)	Inundation mapping Elevation profile Future shoreline location
Tidal surface (MHHW)	NOAA (2023)	Inundation mapping Future shoreline location
Parcel data	County property appraiser offices	Property/shoreline ownership Parcel details
Shoreline characteristics	University of Central Florida (Donnelly et al. 2018)	Shoreline characteristics
Demographic Index	EJScreen (US EPA 2024)	Group representation

To map sea level inundation, we developed an approach based on free and open-source geospatial software. The approach follows the passive inundation mapping processes developed by NOAA (2017) and as described in detail by Juhasz et al. (2023). The land elevation data and corresponding digital elevation model (DEM) were obtained from the most recent LiDAR survey commissioned by the Florida Division of Emergency Management. The DEM has a horizontal resolution of 0.76 m and vertical accuracy of ≤ 0.2 m (Stoker and Miller 2022). In this study, inundation was defined as a land surface located below the mean higher-high water (MHHW) level and hydrologically connected to the estuary. While it is a generally accepted practice in inundation mapping to use a constant value to approximate the MHHW surface, this is not accurate over larger areas since MHHW is spatially variable, especially in estuarine environments. To support accurate, parcel-specific planning, we used VDatum Transformations developed by NOAA (2023) to approximate this variation as described by Juhasz et al. (2023). All horizontal data are referenced to the North American Datum 1983 (NAD83), Universal Transverse Mercator (UTM) zone 17N coordinate system, and resampled to 3 m pixel resolution. All elevation values are expressed in the NAVD88 vertical datum.

The intersection of the MHHW surface and the DEM was defined as the shoreline. The location of future shorelines was mapped using the same DEM and the MHHW surface corresponding to the sea level rise scenario and time step selected by the user. This approach preserves the spatial variability of the MHHW surface but assumes no future change in that variability nor in the topography of the land surface being inundated. For the purposes of this pilot project, only shoreline segments longer than 1500 ft (457 m) were considered. This was done to optimize computational and storage efficiency while minimizing the extent of omitted shoreline segments. In addition to the plan view maps of inundation, a transect attribute option was also created to facilitate visualizations of the extent and location of upland flooding along a shore-normal topographic profile corresponding to the sea level trajectory and time step selected by the user. The elevation along each transect line is based upon the underlying DEM and sampled at 15 ft (5 m) intervals. The slope between adjacent elevation points is automatically calculated and included in the graphical output.

**Fig. 3:**
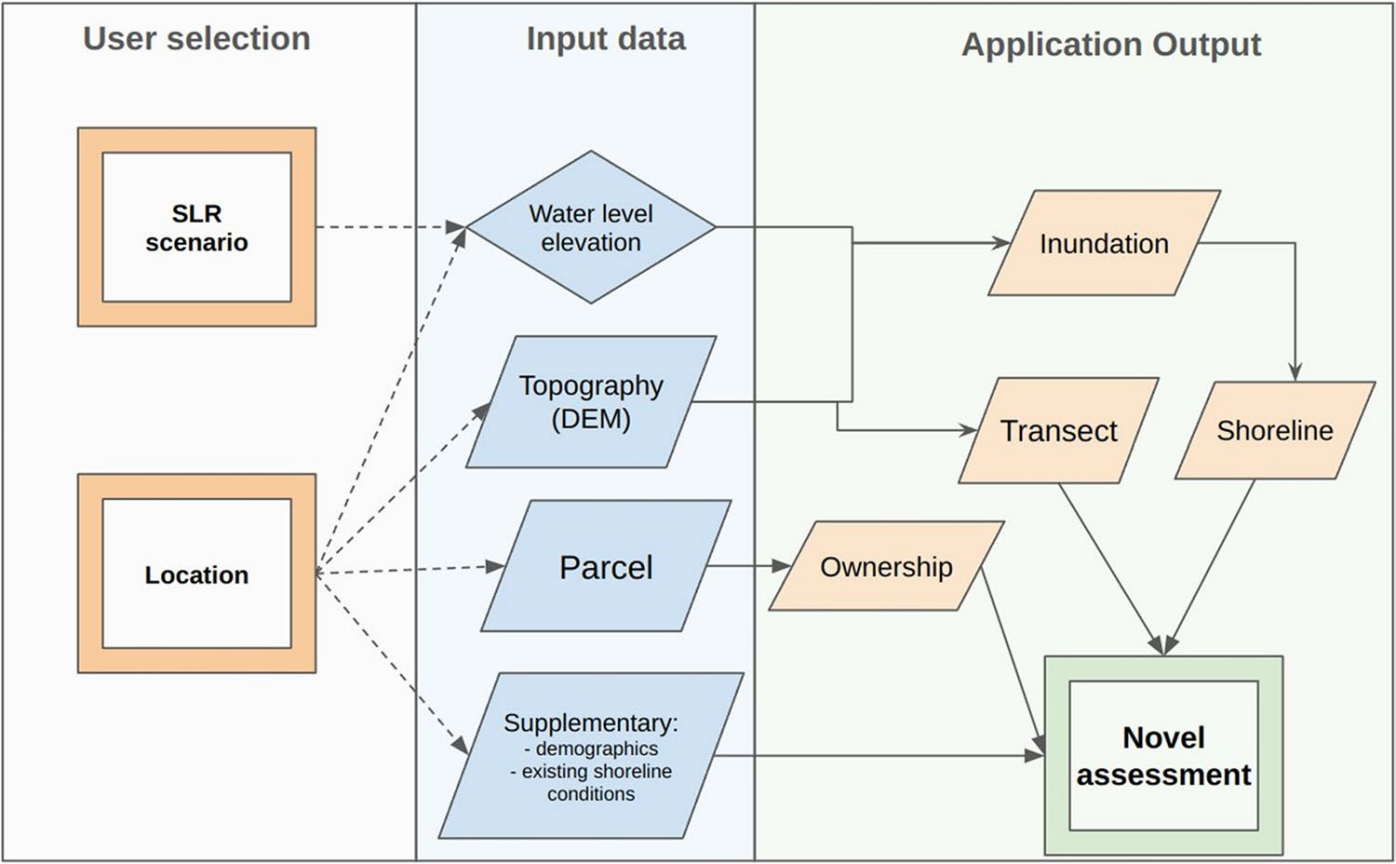
Schematic diagram summarizing the decision framework of the Future Shorelines tool. User selected conditions are combined with input data to generate a novel suite of application outputs that can be used to identify nature-based shoreline stabilization locations that are climate resilient

Parcel data were incorporated into the decision support tool as a map menu option to delineate the ownership and boundaries of properties located along the IRL shoreline. The demographics of people residing in the study domain were also integrated into the model at the census block group level using the EPA Environmental Justice Screening Tool (US EPA 2024). This attribute was included because federally funded coastal zone management programs now generally require applicants who are soliciting support include outputs and outcomes that address social justice inequities. A summary of the tool’s decision framework and outputs is provided in Fig. 3.

### Project Design and Implementation

**Fig. 4:**
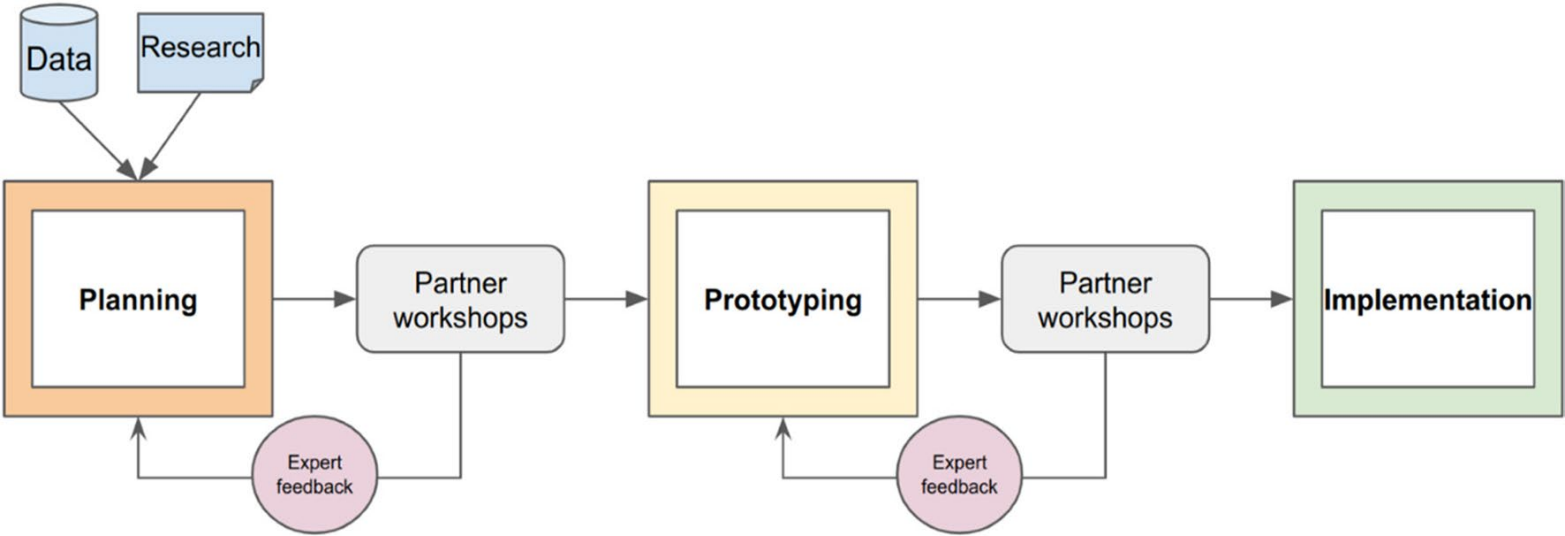
Flowchart illustrating the collaborative, user-centric coproduction process employed during the development and implementation of the Future Shorelines tool

Central to the success of this project was the incorporation of feedback from our partners and other practitioners based upon a philosophy that prioritized end user input and a user-centered design (Pratt and Nunes 2012). This coproduction (Beier et al. 2017) allowed for the refinement of the methodologies and outputs using an iterative process that incorporated end-user feedback and suggestions throughout the development of the application (Fig. 4). More than 40 virtual workshops were held throughout the duration of the 2-year project. The initial workshops focused on presenting an overview of the project and methodological approach, while explicitly seeking feedback on key points in real time, follow-up correspondence, and polling. Subsequent workshops featured prototypes or beta versions of the application that allowed our partners to provide more targeted feedback based upon their experience. As a result, numerous modifications to elements of the project’s original scope and design, including changes related to data, application functionality, and the user experience, were made as summarized in Table 3 and described in the following sections. The use of a coproduction framework yielded a project deliverable that aligns with the needs of the practitioners for whom it was intended and therefore elevated the probability of its use.

**Table 3:** Future Shorelines data and functionality issues resolved by coproduction with project partners

Topic	Issue	Resolution
Units of measure	What unit of measure should be used?	US customary (SAE)
Shoreline profiles	What should be the distance between transects?	100 feet (30 m)
	How far should a transect extend inland?	500 feet (152 m)
Shoreline characteristics	Addition of shoreline characterization data would be helpful	County partners provided shoreline characterization geospatial files
Parcel data	Some of the Florida Department of Revenue ownership and taxed assessed value data were inaccurate	County partners provided parcel data from their property appraisers office
Parcel search	Can parameters other than the parcel ID be used to navigate to a location of interest?	Added physical address and geographic coordinates to the menu of search parameter options
Polygon features	Shoreline ownership designations obscure details of underlying aerial imagery	Created a slide bar so user can adjust the transparency of shoreline ownership designations

#### Changes Related to Data Elements

On-line partner polling at the onset of project development indicated a preference for the use of US customary units of measure to express distance and height because these were more easily integrated into their day-to-day operations. Upon the introduction of the shoreline elevation profile attribute, the project partners were again polled regarding the preferred profile length and alongshore distance between them. Results indicated a preference for a length of 500 ft (152 m) inland from the present shoreline on 100 ft (30 m) center points. A beta version of the application was presented to the project partners during the second workshop that utilized statewide parcel data compiled by the Florida Department of Revenue. They noted that some federal properties were erroneously marked as “land with no value or ownership.” As a result, the statewide dataset was replaced with data collected by each county’s property appraiser and subsequently provided to the project team by them. The ownership categories (i.e., private, public) and other metadata (e.g., parcel ID, class, value) were extracted from these files using a rule-based approach. The project partners also expressed interest in the addition of a point dataset of shoreline characteristics (e.g., seawall, riprap, natural) that had been recently compiled for southern Volusia and all of Brevard County. This dataset was generated by the University of Central Florida (Donnelly et al. 2018) and provided by both counties for integration into the application as an additional mapping attribute.

#### Changes Related to Functionality

The beta version of the application allowed users to search for specific parcels based on their unique identification number (i.e., parcel ID). While useful, the project partners expressed a desire to search for sites using geographic coordinates or a street address. The interactive functionality of the web application was upgraded to include both in the menu of search options.

#### Visual and Contextual Changes

In a Geographic Information System (GIS) framework, visual hierarchy is crucial since spatially overlapping features can obscure information from the user’s view. During the third workshop, our partners noted that the shoreline ownership polylines overlapped the base map and shoreline characteristics dataset, thus obscuring that information. In response, the shoreline ownership feature was modified so that its transparency could be manually adjusted.

### Additional Details on the Methodology

Methodological details about sea level rise inundation modeling, geospatial processing, and development of the technology platform used in this project can be found in Juhasz et al. (2023). Additional methodological information (e.g., digital elevation model, MHHW tidal surface) can be found on the publicly available project landing page (https://futureshorelines.fiu.edu) by selecting the “Publications & Data” tab.

## Results

In the following sections, three examples are presented describing how the living shoreline site selection and design decision support tool (hereafter Future Shorelines) can be used by practitioners to optimize the performance and resiliency of projects subjected to sea level rise in the coming decades. A fourth example is also provided in which the tool’s application to topographic recontouring, an emerging nature-based solution designed to restore wetland habitat in areas impacted by the historical placement of spoil or fill, is described. In all cases, NOAA’s high scenario-based sea level rise trajectory was utilized because twenty-first-century rates of rise along the southeast US coast are currently tracking with the intermediate high and high trajectories and expected to continue accelerating in the decades ahead (Parkinson and Wdowinski 2023a, 2023b).

### Changes in Land Ownership

**Fig. 5:**
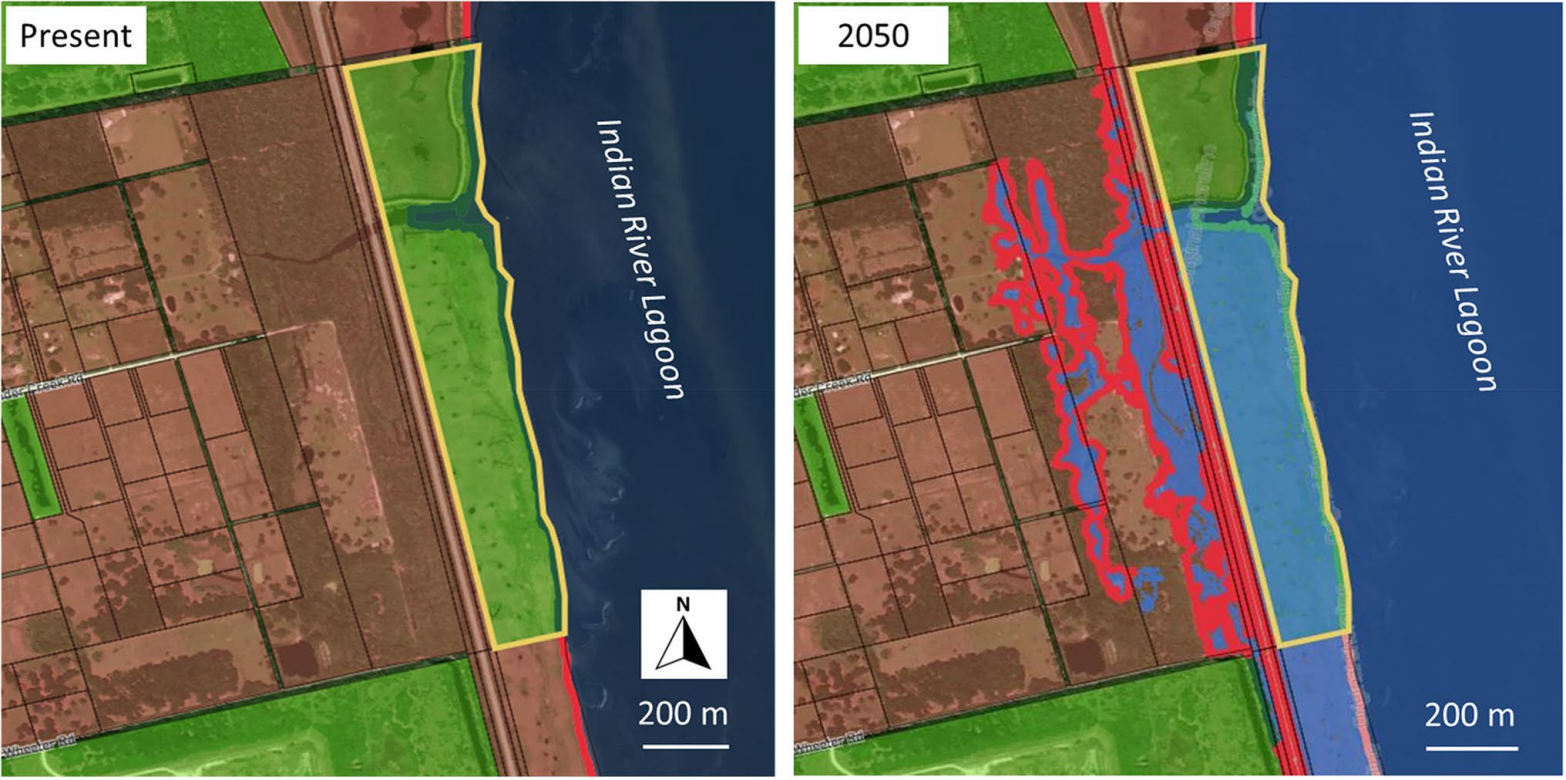
Time series illustrating selected parcel boundary (yellow polygon) and Indian River Lagoon shoreline at present (2020, left) and in 2050 (right). Over the duration of this 30-year interval, sea level rises 1.51 ft (~ 0.5 m) under the High NOAA trajectory. This results in upland submergence and shoreline transgression onto a parcel that is not publicly owned. This may complicate the ability of practitioners to access the site for the purpose of monitoring and maintenance over the design life of the installation. Color legend as follows: blue, water; red line and infill, private shoreline and land ownership; green line and infill, public shoreline and land ownership. See Fig. 1 for location within the study domain

Most of the existing living shoreline installations within the study domain are located adjacent to public land. This association was not unexpected because it is generally easier to negotiate an agreement to construct, monitor, and maintain them at these locations. Hence, in this first example (Fig. 5), a potential installation location was identified wherein the current ownership of an undeveloped shoreline and adjacent upland parcel is within the public domain. However, as the sea level rises, the shoreline at this location will migrate landward, and by 2050, it will abut private land. This may confound the ability to sustain a performance monitoring or maintenance program should the installation still be functional at that time. With this knowledge in hand, the practitioners have the option to resume their search using the Future Shorelines tool to identify alternate locations where this change in ownership is not likely to occur.

### Installation Resilience and Allocation of Practitioner Resources

**Fig. 6:**
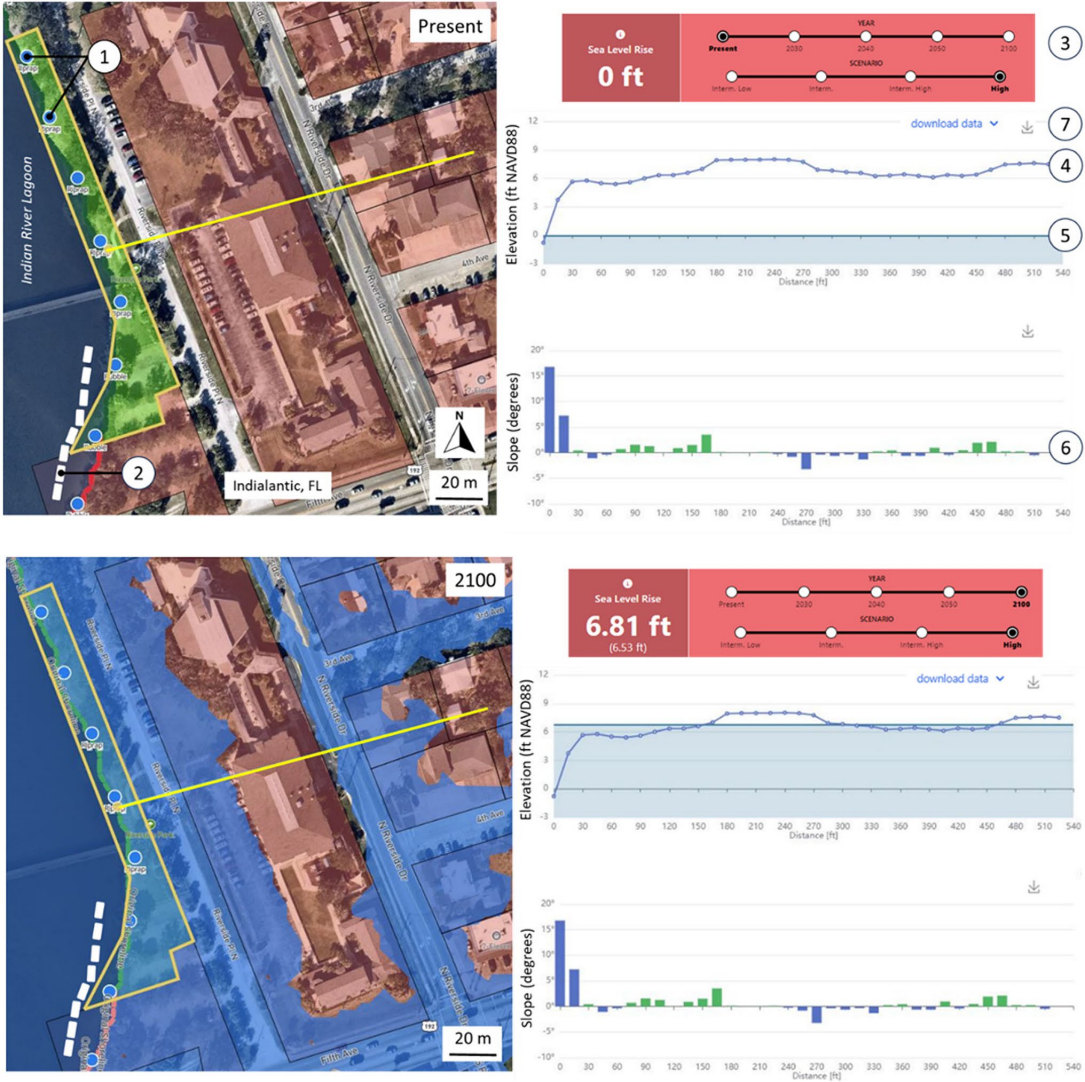
Left panel. Time series illustrating location of shoreline and submerged lands at present (2020, top) and in 2100 (bottom). Also shown are shoreline characterization observations provided by the University of Central Florida (Top, 1) and the location of an existing oyster reef installation or breakwater (Top, 2). Right panel. Output of time steps corresponding to the high NOAA sea level rise scenario at present (Top, 3) and in 2100 (Bottom). Corresponding to each time step are paired elevation (4, land; 5, MHHW) and land slope (6) values generated along the selected 500 ft elevation transect (left panel yellow line). Slope values organized into two categories to distinguish upland areas considered too steep for the successful landward migration of a living shoreline (> 5°, blue histogram bars) and those more favorable (< 5°). See text for details. Options to download the raw elevation data in a KML or CSV format or the transect image as a png file are also available (7). Color legend for left panel as follows: blue, water; yellow polygon, parcel boundary; red line and infill, private shoreline and land ownership; green line and infill, public shoreline and land ownership. See Fig. 1 for location within the study domain

In this example, a location was identified where a living shoreline project was recently constructed along a narrow, publicly owned shoreline parcel (Fig. 6 left panel). It consists of oyster breakwater and mangrove plantings located adjacent to a hardened shoreline of riprap that ascends steeply (i.e., ~ 16°) to an inland elevation of 6 ft (1.8 m). The intended ecosystem services of this installation are not expected to persist under conditions of sea level rise because the shoreline is too steep. This expectation is based on observations that indicate the most extensive and persistent coastal wetlands in Florida are located in areas where the landscape slope is < 5° (e.g., Everglades, Big Bend). In these areas, wetlands have survived for centuries to millennia under conditions of sea level rise by (1) migrating inland at the same rate as the transgressing shoreline and (2) vertically accumulating soil to remain within the intertidal zone (cf. Parkinson and Wdowinski 2022, 2023a). Because the slope of the shoreline at this location is ~ 16°, sea level rise will create relatively limited horizontal accommodation space into which the existing wetlands can migrate. In 2050, for example, sea level will have risen 1.51 ft (0.46 m) relative to 2020 and the shoreline will have migrated 5.5 ft (1.7 m) upslope. By contrast, if the slope was 5°, the shoreline would have migrated inland about 17 ft (5 m), creating a much broader area into which the installation’s mangrove plant community could migrate. Given the limited capacity to migrate inland at this location, the resiliency of this living shoreline is therefore primarily dependent upon its capacity to aggrade vertically at the same pace as the rising seas. These observations are informative because they underscore the value in selecting installation construction locations that are adjacent to low-relief shorelines and upland areas. The Future Shorelines tool provides a graphical depiction of the shoreline slope along the topographic profile selected by the user (Fig. 6 right panel). The slopes are subdivided into two categories based upon the 5° threshold value (see also Mitsova and Bergh 2016) and can be used to quickly assess the resiliency of a nature-based installation at any location of interest in the context of the availability of horizontal accommodation space.

The resiliency prognosis of this installation can also be used as an additional means of decision support in the context of resource allocation. Practitioners responsible for the monitoring and maintenance of living shorelines are typically constrained by the availability of human and financial capital (cf. Kirschke et al. 2020). The Future Shorelines tool provides a platform in which the practitioner can conduct a systematic evaluation of the installations they manage to gauge the extent to which rising seas will yield a contraction or expansion of intertidal substrate into which the wetlands can migrate. The outcome of that exercise can then be used to prioritize monitoring and maintenance efforts at living shoreline installation locations where the conditions favorable to tidal wetland colonization will not diminish as sea level rises.

### Environmental Justice

**Fig. 7:**
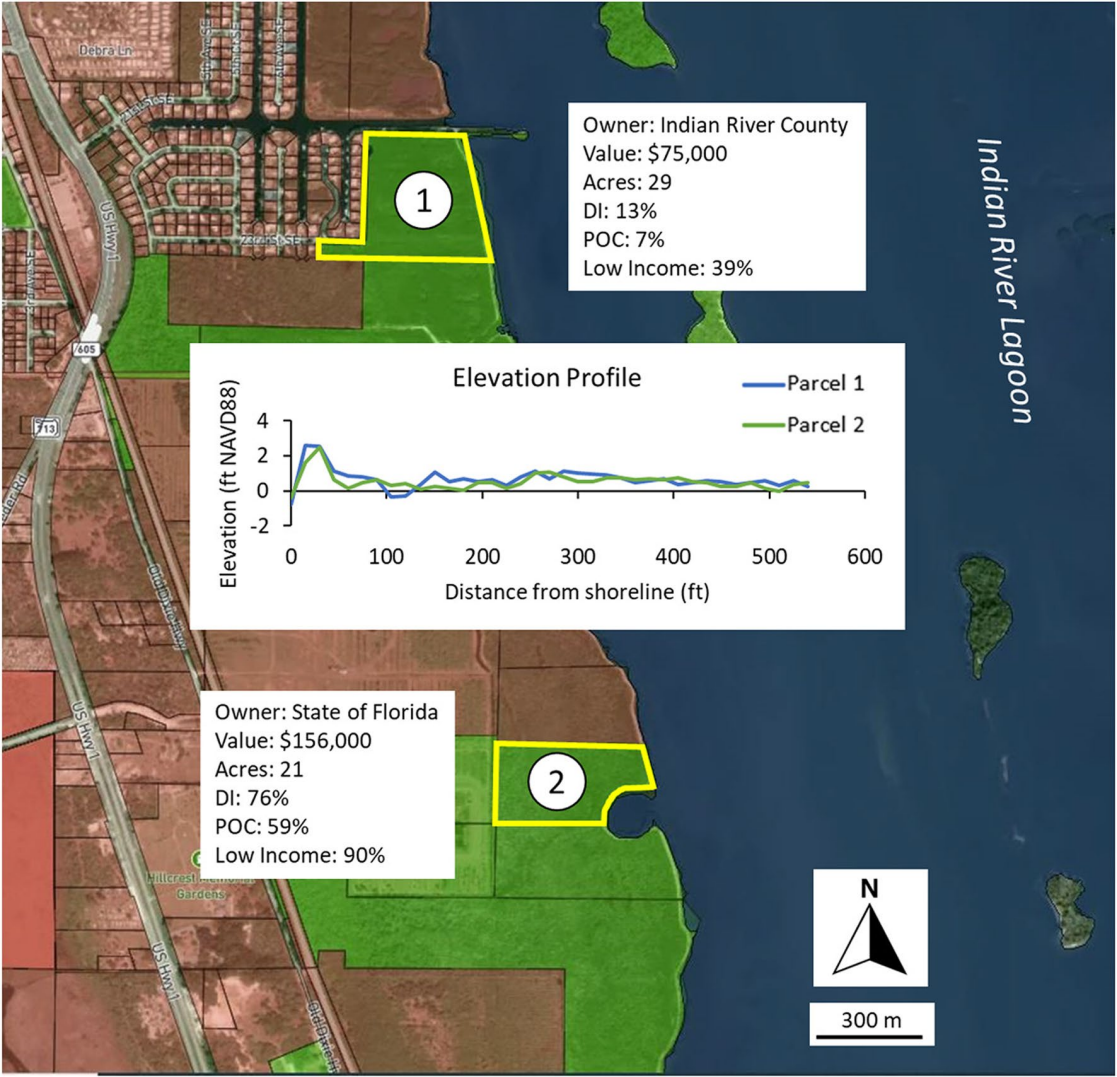
Example of Future Shorelines model output to address issues of environmental justice. Parcels 1 and 2 are similar with respect to ownership, land value and size, and elevation. The two are distinguished by the EPA Demographic Index (DI) census block score. Parcel 1 is scored at 13% and parcel 2 at 76%. A project constructed along the shoreline of parcel 2 would be preferable if the intended project outcomes include the desire to address environmental justice inequities. Color legend as follows: blue, water; yellow polygon, parcel boundary; red infill, private shoreline and land ownership; green infill, public shoreline and land ownership. See Fig. 1 for location within the study domain

The US government now requires grant applicants soliciting federal funds to mitigate or restore the coastal environment include an environmental justice component in their proposed scope of work (e.g., outputs or outcomes). Our application utilizes a demographic index (DI) attribute to assist practitioners in meeting this requirement. The index was obtained from the EPA’s on-line EJScreening Mapping Tool (US EPA 2024) and is graphically displayed as a mosaic of census block group layers distinguished by their DI score. The score ranges from zero to one and is calculated based on the percentage of the population self-identified as people of color and low-income census block score (i.e., [% people of color + % low income]/2). In the example provided (Fig. 7), two coastal parcels were selected that are similar in land area, value, and elevation. However, the DI scores are significantly different at 13% (parcel 1) and 76% (parcel 2). Construction of a living shoreline project along the shoreline of parcel 2 would be preferable if an intended outcome includes consideration of underserved communities who may not otherwise benefit from the ecosystem services they provide. This could be facilitated by soliciting local resident participation in pre-construction meetings and assistance during the construction, monitoring, and maintenance phases of the project.

### Shoreline Recontouring

**Fig. 8:**
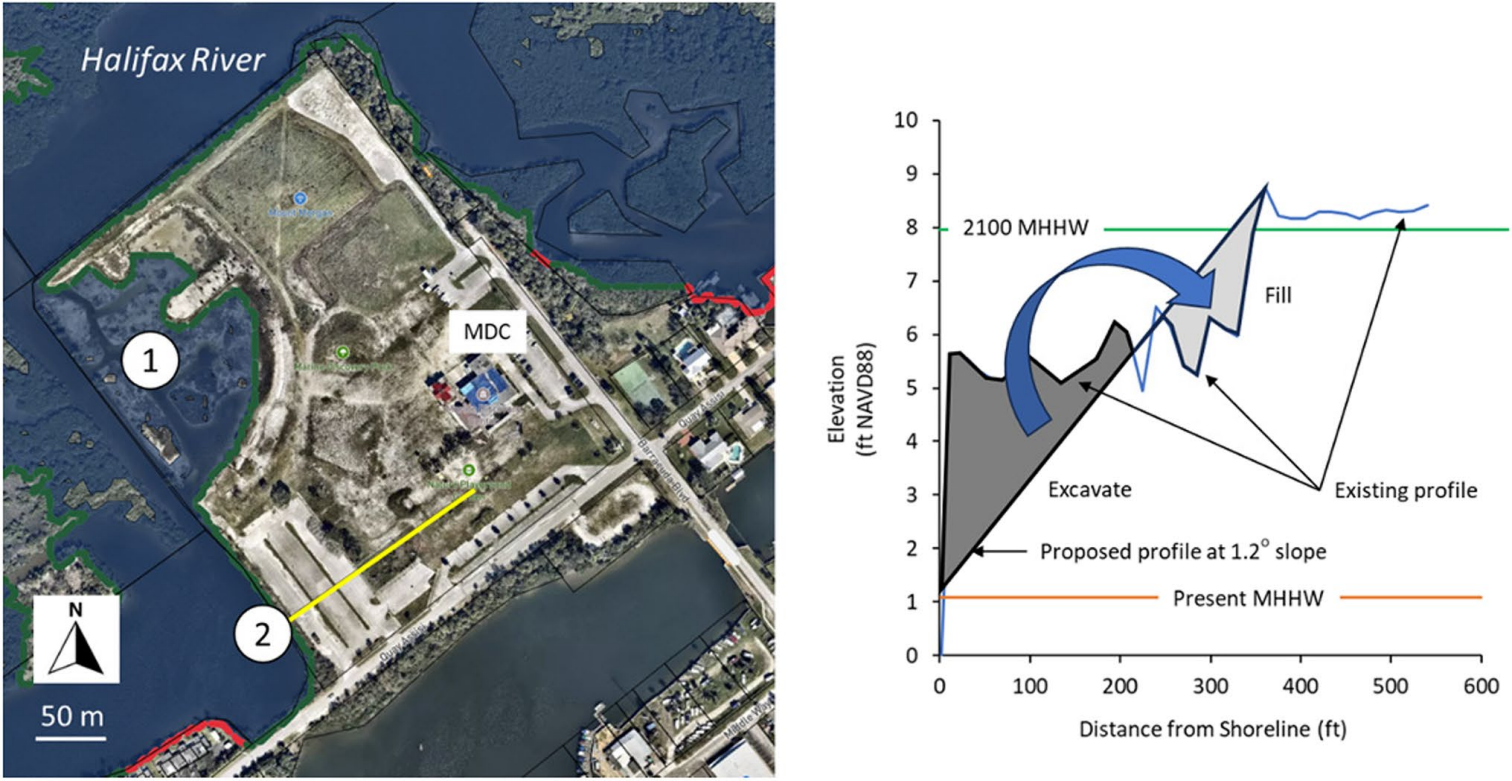
Left panel. The Marine Discovery Center (MDC) has successfully recontoured the topography of an area of fill to elevations that now support the full assemblage of wetland plant communities (1) and is currently considering a second project (2). Color legend as follows: red line, shoreline privately owned; green line, shoreline is publicly owned; yellow line, location of elevation profile. Right panel. Elevation profile of the existing landscape and MHHW level in year 2100 constructed using data downloaded from Future Shorelines tool in support of a preliminary design analysis of a proposed topographic recontouring project. In this example, the hypothetical goal was to achieve an acceptable shoreline grade and optimize the beneficial use on on-site spoil to create a restoration site with a design life of 80 years (i.e., 2020–2100). The existing 1.2° slope could be modified to create conditions in which the volume of fill excavated is equal to the volume of fill needed. See Fig. 1 for location within the study domain

An emerging element of coastal restoration is the targeting of areas where fill or spoil was placed during historical times (e.g., construction of the Intracoastal Waterway) for topographic recontouring to elevations conductive to wetland restoration (cf. Robison et al. 2020). The Marine Discovery Center (New Smyrna Beach, Florida) recently completed a spoil recontouring project on their property to create an intertidal surface that now hosts wetland plant communities (Fig. 8 left panel) and they are currently considering a second recontouring project. Depending on the scale of the proposed shoreline restoration project, the tool provides as many as 10 elevation profiles that can be used during the preliminary design phase to evaluate potential topographic recontouring options with respect to shoreline slope and the beneficial use of on-site materials. In this example, the elevation profile was created along one of the transects using the Future Shoreline’s data download option and graphed as a line extending from the present shoreline to the MHHW elevation in 2050 (Fig. 8 right panel). The slope of the recontoured shoreline is 1.2°. The slope may later be adjusted, so long as it remains < 5° for reasons described previously, to balance the volume of on-site excavation and infill requirements. This beneficial use of spoil is encouraged by the US Army Corps of Engineers (Wilber 1992; Ray 2007). The restored shoreline could then be populated by native wetland and upland plants that would over time migrate up-slope and onto areas that were not targeted for recontouring.

## Discussion

It is important to note that living shorelines are built for a wide variety of reasons, including (1) demonstration projects designed to foster public interest, education, and outreach, (2) projects constructed at the request of a landowner who is interested in nature-based solutions as an alternative to traditional hard structures, and (3) as a soft structural alternative to the repair or replacement of an existing seawall, bulkhead, or riprap shoreline protection feature. In these instances, the intended output is the construction of a nature-based shoreline stabilization feature and the outcome enhanced ecosystem services. Considerations regarding the long-term viability or resilience of these types of projects to sea level rise are of secondary importance or may not have been considered at all. For example, many of the existing IRL living shorelines were constructed as demonstration projects in front of a vertical seawall or steeply aggrading riprap (cf. Figure 6). These are unlikely to keep up with sea level rise in the coming decades as previously described. However, in these examples, the expected output and outcome were achieved immediately upon completion of the project. Therefore, these types of projects are considered successful because they performed as intended. But if the goal of nature-based solutions in general, and living shorelines in particular, is to mitigate historical losses in ecosystem function, habitat value, and water quality impairment, coastal practitioners must begin to consider their longevity or resilience under conditions of climate change and concomitant sea level rise. It was in this context that the Future Shorelines tool was developed. As demonstrated, it provides a suite of menu options that can be used to facilitate the planning and construction of nature-based shoreline stabilization solutions that (1) are located where opportunities for horizontal migration are optimized, (2) remain accessible for monitoring and maintenance, and (3) perform as intended over the design life of the installation. Future Shorelines was constructed using a flexible framework that can be readily populated with other geospatial data or attributes as may be requested by the practitioner (e.g., stormwater outfalls, MHW vertical datum). It can also be seamlessly customized for use in other regions where the requisite data are available.

During the last quarter of the project, our partners were asked to suggest additional data or attributes that could be incorporated to enhance the utility and use of the Future Shorelines tool. These generally fell into two groups, those that facilitated (1) compliance with State (i.e., FDEP) or Federal (i.e., USACE) statutory/regulatory requirements (e.g., alternate sea level rise scenarios and time steps) and (2) project permitting (e.g., alternate elevation datum, bathymetry, seagrass maps). Many of these are included in Table 1 under the category “next generation” and the project team is currently working to acquire funding to incorporate them into the current version of Future Shorelines.

## Conclusions

This paper summarizes the rationale and function of a novel living shoreline site selection and design decision support tool that was developed for coastal practitioners interested in constructing nature-based solutions that are climate resilient. The product, termed Future Shorelines, models sea level rise, coastal submergence, and shoreline translation. When this information is considered in tandem with the parcel data (one of several map menu options), the output provides a novel basis for evaluating potential changes in ownership that may hamper efforts to monitor and maintain an installation. The tool also provides a means of selecting installation locations where rising seas will not contract, but rather expand intertidal areas suitable for wetland colonization as sea level rises. A demographic menu option is available to provide the practitioner with information relevant to site selection in the context of environmental justice. Finally, the tool’s elevation and shoreline profile features can be used to assist in the design of topographic recontouring projects undertaken to restore shorelines and coastal habitats degraded by historical fill or spoil deposition. Future Shorelines benefited greatly from coproduction with the project’s partners, who have recommended additional data and attributes that could be incorporated to further enhance its utility and use during the design, permitting, and construction of nature-based solutions that are climate resilient. These are currently being evaluated by the project team.
